# Basalt Fibre-Reinforced Polymer Laminates with Eco-Friendly Bio Resin: A Comparative Study of Mechanical and Fracture Properties

**DOI:** 10.3390/polym16142056

**Published:** 2024-07-18

**Authors:** Devmith Kariyawasam Don, Johannes Reiner, Matt Jennings, Mahbube Subhani

**Affiliations:** School of Engineering, Deakin University, 75 Pigdons Road, Waurn Ponds, VIC 3216, Australia; devmith.k@deakin.edu.au (D.K.D.); johannes.reiner@deakin.edu.au (J.R.); mahbube.subhani@deakin.edu.au (M.S.)

**Keywords:** basalt fibre, composites, bio resin, fracture, open-hole strength, shear

## Abstract

Fibre-reinforced polymers (FRPs) are widely used in industry due to their impressive strength-to-weight ratio, corrosion resistance and high durability. One of the primary components of FRPs is synthetic resins, specifically epoxy, which has been identified as harmful to the environment. To address this concern, an eco-friendly alternative made from basalt fibres and bio resin has the potential to reduce the environmental impact. This study investigates Basalt Fibre-Reinforced Polymer (BFRP) laminates manufactured using two bio resins, AMPRO™ BIO and Change Climate, comparing them to one conventional epoxy resin, WEST SYSTEM^®^, in terms of tensile modulus, strength and fracture toughness, as well as shear properties. The results indicate that BFRP laminates made with bio resins exhibit comparable or better mechanical properties to their conventional counterparts with tensile strength being between 6 and 17% more in bio resins compared to the conventional resin, thereby paving the way for further exploration of sustainable FRP laminates in future engineering applications.

## 1. Introduction

Fibre-Reinforced Polymers (FRPs) are widely used throughout the world in many industries ranging from automotive to aerospace. FRPs have very high tensile strength and stiffness-to-weight ratios. The commonly used fibres are carbon, glass, aramid and basalt which are usually used with thermoset resins, such as epoxy resin, to produce FRP laminates. Around 95% of products made from carbon fibre composites end up in landfills and are not bio-degradable [1]. The energy required to produce epoxy resins also significantly contributes to greenhouse gas emissions [2]. In order to produce sustainable alternatives that align with the concept of a circular economy, end of life use/process should be taken into account while exploring new materials.

Basalt fibre is a naturally occurring material which is extracted from volcanic rock and is environmentally neutral [3]. This makes it an ideal candidate for the manufacturing of eco-friendly FRPs and can therefore be used as an alternative for the reinforcement of polymers [4]. Similarly, there is potential to replace traditional synthetic resins with bio resins to improve the environmental footprint of BFRP laminates. However, the development of bio resin is an active field of research and 100% bio resin that can meet structural requirements is rare [5]. Development of bio resin is reported in [6,7,8,9]; however, their use in fibre-reinforced polymer, especially for basalt, is not available.

Johnson et al. [10] stated that pure epoxy bio resin (provided by Supreme Silicons Pvt Ltd.) has superior tensile strength compared to the pure polyester and pure epoxy resin that are commercially available on the market. However, long-term performance was not investigated in the study. Also, only tensile tests were performed in the study, where the bio resin was found to have superior performance compared to traditional pure epoxy and traditional pure polyester specimens. In addition, it can be noted that only the properties of the resin were investigated in [8] without considering any fibre reinforcements.

Another study involving bio resins used hemp fibres as the reinforcement [11]. In the study, two bio resins (Greenpoxy 55 and super sap 100/1000) and one traditional resin (SP 110) were compared in terms of tensile and flexural properties. The result suggested that the performance of bio resins is slightly lower compared to traditional resins. The study also stated that there were challenges with vacuum infusion using bio resins due to its high viscosity.

Various research has been undertaken to produce partial or full bio-based composites [12]. To this endeavour, mechanical performance of natural fibre along with traditional epoxy received more focus than bio-based epoxy with natural fibre. Several studies have investigated the use of plant-based natural fibres such as harakeke and bamboo with epoxy resin as a low-cost ecofriendly composite solution using different variables, and the results usually indicate acceptable mechanical properties [13,14,15,16], some of which even challenging established composites such as glass/epoxy composite laminates [17]. One of the hurdles that plant-based natural fibres have to overcome is the hydrophilic nature of plant fibre and the hydrophobic nature of the epoxy matrix which reduces the bond between the two. Studies have been conducted to improve this bond using several chemical treatments on the natural fibre surface [18,19,20,21,22,23]. To improve the mechanical properties and moisture resistance of the plant based natural fibres some studies aimed for hybridization with mineral-based natural fibres such as Basalt Fibres. According to et al. F.A. Almansour, in a study which uses flax–basalt hybrids, the specimens without basalt hybridization absorbed higher moisture; the study also states that the external skin layers of basalt fibres exhibited a greater resistance to water absorption and prevented the inner core of flax fibres from degradation [24].

There are several studies regarding mode I and II fracture toughness, one such study examines the effects of nanoparticles on nanocomposites and its effects on mode I and mode II fracture characteristics [25]. Another study had a hybrid structure with a cork core as opposed to pure basalt fibre, and compression and flexural properties were evaluated [26]. Limited studies of fracture properties of basalt fibre with traditional epoxies are reported for mode I in [27,28] and for mode II in [27]. Studies on BFRP laminates with bio resin in terms of interlaminar fracture toughness properties are absent.

The main goal of this study is to evaluate the mechanical and fracture properties of laminates reinforced with basalt fibres using commercially available bio resins, compared to a cost-effective high-usage fossil-based epoxy resin. Two types of bio resins were considered in this study with 40% and 77% bio-based content. The following properties were determined and compared: tensile properties, open-hole tensile strength, shear properties and mode I and II fracture toughness. The results were then compared to the literature to determine the future viability of the materials as a potential candidate to produce more sustainable laminates.

## 2. Materials and Methods

The mechanical performance of the BFRP laminates were tested as per the ASTM standards. The following test standards were utilised: tensile testing of pristine samples (ASTM D3039/D3039M), open-hole tension test (ASTM D5766/D5766M), ±45 shear testing (ASTM D3518/D3158M), mode I interlaminar fracture toughness (ASTM D5528–13) and mode II interlaminar fracture toughness (ASTM D7905/D7905M–19).

### 2.1. Sample Preparation

Biaxial basalt fibre fabrics of 600 g/m^2^ (Product code DB600 from Basalt Fiber Tech, Oakleigh, VIC, Australia) were used to make laminate panels with dimensions of 500 mm × 350 mm. Four biaxial layers of fabric were stacked to create a laminate with a total thickness of approximately 2.5 mm to meet ASTM standard requirements. The chemical composition of the components of basalt fibre (as % weight) used in this study were as follows: SiO_2_ (54.5–55.55%), MgO (4–4.6%), CaO (7.5–8.5%), Fe_2_O_3_ + FeO (10–11.5%), Al_2_O_3_ (16.5–18.0%), TiO_2_ (0.9–1.25%), ΣR_2_O (4–5%) and Li_2_O (0.1–0.3%) [29].

The epoxy resins used are listed in Table 1. As per the manufacturer datasheet about the composition of the resin, some of the ingredients are bio-based (plants), while others are sourced from hydrocarbons. The amount of ingredients sourced from plants and hydrocarbon varies, and hence the AMPRO™ BIO (Yatala, QLD, Australia) and the Change Climate (Adelaide, SA, Australia) epoxies can be considered as 40% and 77% bio-sourced, respectively. The tensile strength of the neat epoxies made from WEST SYSTEM^®^(Bay City, MI, USA), AMPRO™ BIO and Change Climate were reported by the manufacturer as 50.3 MPa, 36.3 MPa and 54 MPa, respectively. The tensile modulus of the same three epoxies were 3.2 GPa, 1.9 GPa and 4.5 GPa, respectively [30,31,32].

The target resin-to-fibre ratio in this study was 50/50. Each stack of dry fabric was weighed prior to adding resin, and the same amount in weight of resin was mixed. A wet layup process was adopted to impregnate the dry basalt fibre fabrics using the aforementioned resin systems. A thin Teflon film on a flat aluminium plate enables the release of the laminates after curing. First, a layer of resin was applied on the release film followed by placing the first layer of basalt fibre fabric. A squeegee and a roller were used to wet the basalt fabric. Once the fabric was wet, more resin was applied, and this process continued until all four layers were placed. A peel ply was laid on top of the stack to absorb excess resin and create a smooth top surface. The panel was then left to cure in a fume hood for 24 h.

**Figure 1:**
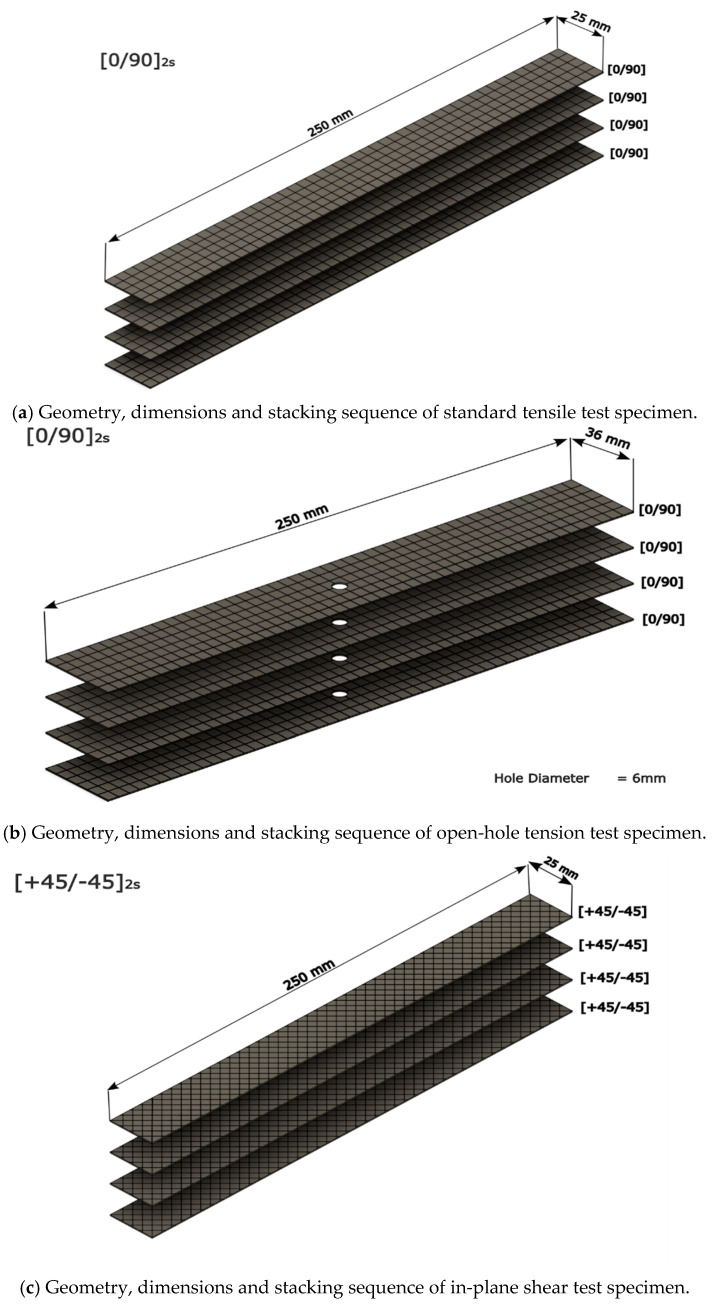

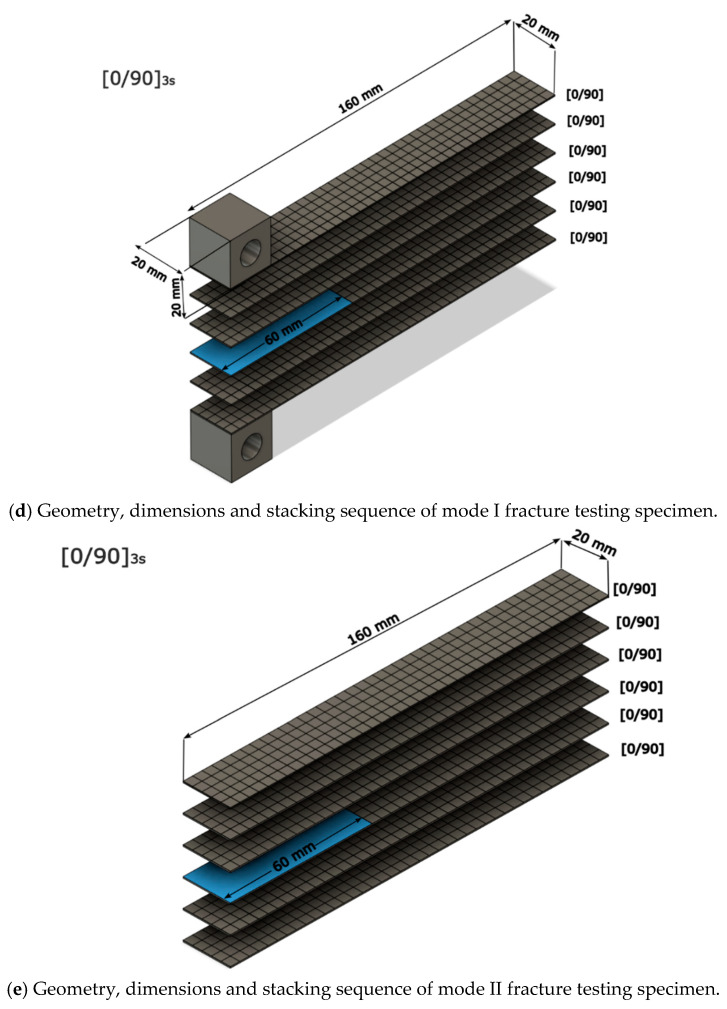
Illustrations of test samples investigated in the present study.

The stacking sequences for various testing cases were as follows, which is also shown in Figure 1a–c:

- For standard tensile tests, [0 90]_2s_;
- For open-hole tensile tests, [0 90]_2s_ with 6 mm diameter hole at the centre;
- For standard in-plane shear tests [±45]_2s_.

Laminated composites are often susceptible to delamination. Delamination can occur in tension (mode I) or under shear (mode II) at the interface, as well as mixed forms. Therefore, it is important to measure the materials resistance against delamination which can be determined in interlaminar fracture toughness tests. In the present study, both mode I and mode II testing were performed to obtain interlaminar properties of the basalt fibre composites for all three resin systems. The stacking sequence for these tests was [0 90]_3s_, as shown in Figure 1d–e.

After the laminates were cured, the test samples were cut to size as per the ASTM standard using a water jet cutter (OMAX Waterjet 55100, Kent, DC, USA). A summary of all the tests is listed in Table 2.

### 2.2. Tension and Shear Properties

**Figure 2:**
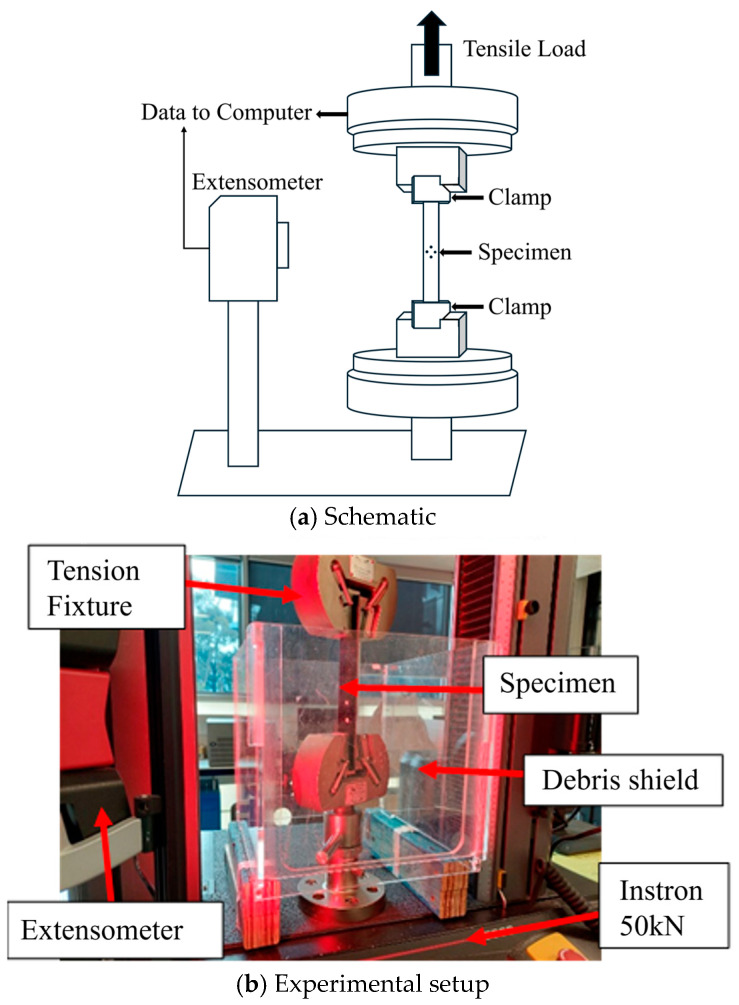
Test set up for tension, open-hole tension and in-plane shear testing. Schematic was adapted from Ng and Hu [40].

To conduct tension, open-hole tension and in-plane shear tests, a 50 kN Instron universal testing machine was used (Instron 5969, Norwood, MA, USA). The test setup is shown in Figure 2. The tests were conducted under displacement control with a rate of 2 mm/minute until the specimen failed. The local strain was measured using a video extensometer by tracking two points from the expected area of the fracture.

The obtained data from the tests enable the calculation of the ultimate tensile strength $\left(F^{tu}\right)$, ultimate open-hole tensile strength $\left(F_{x}^{OHTu}\right)$ and maximum shear stress $\left(\tau_{12^{m}}\right)$. Tensile moduli were determined from the slopes of the tensile stress vs. strain curves between 10 and 40% stress level.

Open-hole ultimate strength was determined according to ASTM D5766/D5766M such that

(1) $$F_{x}^{OHTu}=\frac{P^{max}}{A}$$

where $P^{max}$ is the maximum force before failure and *A* is the cross-sectional area (neglecting the hole in the centre of the test sample).

To plot the shear stress vs. strain graph, load values were converted to shear stresses via

(2) $$\tau_{12i}=\frac{P_{i}}{2A}$$

where $\tau_{12i}$ is the shear stress at the *i-th* data point, $P_{i}$ is the Force at the *i*-th data point and the maximum shear stress $\tau_{12^{m}}$ refers to the highest shear stress. According to Lisle, T. et al. [38], The shear strain, $\gamma_{12i}$, is calculated as

(3) $$\gamma_{12i}=\varepsilon_{xi}-\varepsilon_{yi}=\left(1+v_{xy}\right)\varepsilon_{x}$$

where $\varepsilon_{xi}$ is the longitudinal normal strain, and $v_{xy}$ is the homogenized total Poisson’s ratio (for basalt fibre laminates $v_{xy}$ = 0.3 [39]).

Shear modulus (shear chord modulus, $G_{12}^{chord}$) was determined from shear stress vs. strain plots between the range of 2000 and 6000 micro-strains, με, as per the ASTM Standards.

(4) $$G_{12}^{chord}=\frac{\Delta \tau_{12}}{\Delta \gamma_{12}}$$

where $\Delta \tau_{12}$ is the difference between the two points of applied shear stress, and $\Delta \gamma_{12}$ is the difference between the two points of shear strain.

### 2.3. Mode I Interlaminar Fracture Toughness

**Figure 3:**
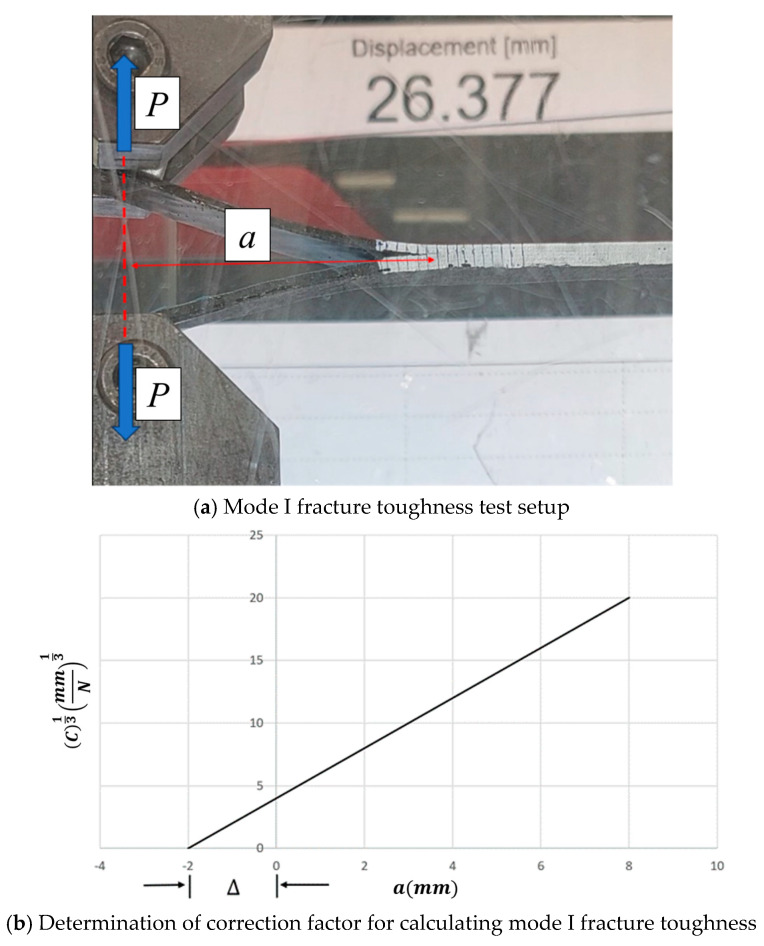
Test setup and calculation of mode I fracture toughness.

For Mode I interlaminar fracture toughness tests, a 10 kN Instron universal testing machine was used (Instron 5966, Norwood, MA, USA). The initial crack length was 60 mm which was created by placing a thin Teflon sheet as shown in Figure 1d. Vertical lines were drawn at every 2 mm from the initial crack tip, spanning over a length of 30 mm, as depicted in Figure 3a. A tensile load was applied on the fixture shown in Figure 1d at a rate of 2 mm/minute until the crack propagated to a total length of 75 mm.

Modified Beam Theory (MBT) calculates the mode I strain energy release rate $\left(G_{I}\right)$ as described in ASTM D5528–13 such that

(5) $$G_{I}=\frac{3P\delta }{2b\left(a+\left|\Delta \right|\right)}$$

where *P* is the measured load, δ is the measured displacement and *b* represent the specimen width; *a* is the delamination length, and Δ is the correction factor. This correction factor was determined by using the intercept of the compliance graph where compliance, *C*, can be calculated as δ/P. The cube root of compliance, *C*, is then plotted against delamination length, a, to find the intercepting point between the two to determine the value of Δ. Figure 3b shows the calculation of Δ. A conventional mobile camera was used to record the entire test so that the delamination length could be correlated to the measured displacements.

### 2.4. Mode II Interlaminar Fracture Toughness

**Figure 4:**
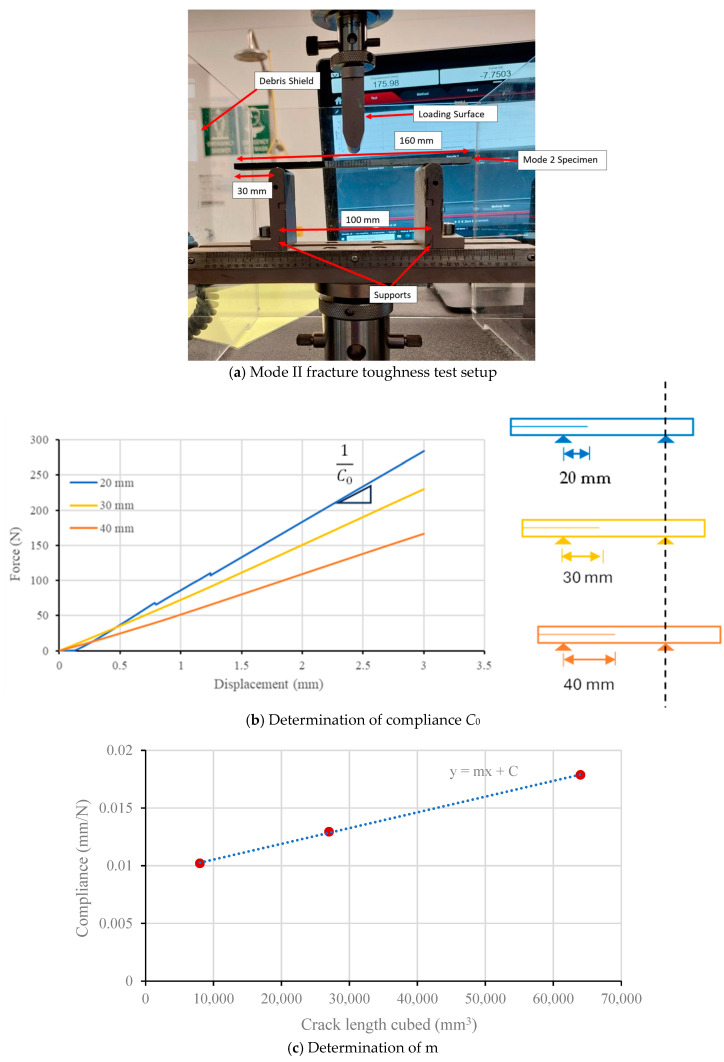
Test setup and calculation of mode II fracture toughness.

Mode II interlaminar fracture toughness test was conducted with an end notch flexure (ENF) test, subjected to 3-point bending. The span length for the mode II samples was 100 mm, while the total span length was 160 mm. Similar to the mode I tests, the initial crack length was 60 mm and vertical lines were drawn from the crack tip at every 2 mm over a length of 30 mm. These samples were tested using the 10 kN universal testing machine. The tests were carried out at a rate of 0.5 mm/minute. Figure 4a displays the test set up for mode II fracture testing.

Since tracking of crack propagation is more challenging in mode II, the compliance-based method was employed to calculate the initial fracture energy $G_{II_{c}}$ which does not require the monitoring of the crack length. According to ASTM D7905/D7905M–19, the fracture energy in mode II interlaminar fracture tests can be calculated such that

(6) $$G_{II_{c}}=\frac{3mP_{Max}^{2}a_{0}^{2}}{2b}$$

where *P* is maximum force, $a_{0}$ is the initial crack length, *b* is the specimen width and *m* is the slope obtained from compliance vs. crack length cubed data.

To determine the compliance, $C_{0}$, a sample with three different initial crack lengths was tested, as shown in Figure 4b. For each initial crack length (20, 30 and 40 mm were considered in this study), force vs. displacement curves were determined, and the inverse slope of the curve can be considered as the compliance $\left(1/C_{0}\right)$. These three compliance values were then plotted against the crack length cubed data (Figure 4c), and the slope of the $C_{0}\;\mathrm{vs}.\;a_{0}^{3}$ is *m*, as outlined in Equation (6).

## 3. Results and Discussion—Tension and Shear

### 3.1. Tensile Properties

All samples of the uniaxial tension test failed due to fibre failure. The location of fracture can be broadly categorised in two groups.

1. Failure near the grip of the specimen;
2. Failure within the gauge area (middle part of the specimen).

**Figure 5:**
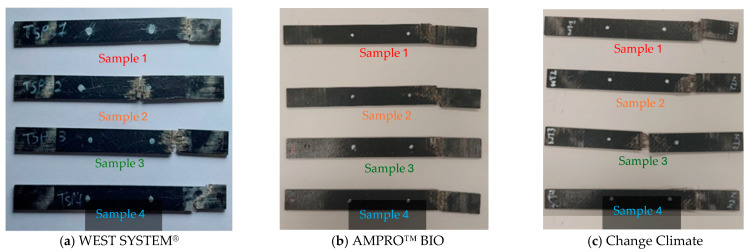
Failure modes of tensile coupons of BFRP laminates with various adhesives.

**Figure 6:**
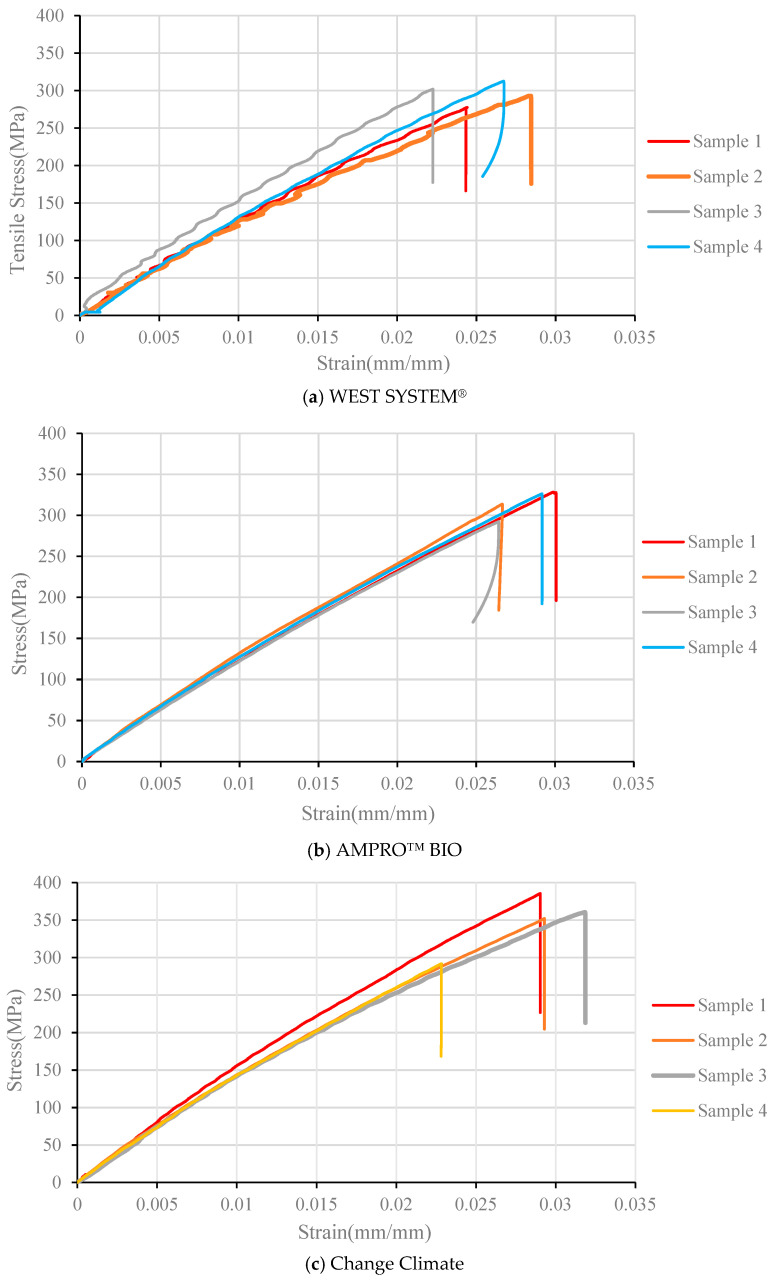
Tensile stress vs. strain curves of BFRP laminates with various adhesives.

In all three resin systems, the first failure location is dominant, whereas the second failure location can only be observed twice (one from WEST SYSTEM^®^ and one from Change Climate), as clearly visible from Figure 5. However, since the failure was governed by the fibre fracture and not bearing, it did not affect the tensile stress vs. strain behaviour greatly, as observed in Figure 6.

Figure 6 compares the tensile stress vs. strain plots of all the tensile samples with three different adhesives. The results are consistent in terms of the slope (modulus) of the curves and ultimate tensile strength. Sample 4 in Figure 6c, made from Change Climate epoxy, was found to have significantly lower (21% lower than the average of the other three samples) tensile strength. This is due to a change in failure mode. As shown in Figure 5c, visible fibre fracture was not observed in that sample. Local crushing (bearing) at the gripping region is visible which led to lower tensile strength.

While comparing Figure 6a–c, the bio resins were found to outperform the traditional epoxy slightly in terms of tensile strength. The AMPRO™ BIO-based BFRP laminates attained 6% higher tensile strength compared to its traditional counterparts, while this improvement was 17% for the Change Climate-based BFRP laminates. Table 3 lists the tensile strength and modulus values of all the samples. It can be noted that the tensile strength of WEST SYSTEM^®^ (WS), AMPRO™ BIO (AB) and Change Climate (CC) were reported as 50.3, 36.3 and 54 MPa, respectively. Therefore, AMPRO™ BIO actually had lower tensile strength than traditional epoxy, yet it yielded 6% higher tensile strength than the BFRP laminate.

The differences in tensile modulus of the BFRP laminates were less affected by the adhesive, as expected. Both the tensile strength and modulus of FRP laminates are mostly governed by the properties of the fibres, rather than those of the matrix. The AB epoxy had slightly lower modulus, whereas the CC had slightly higher modulus than the traditional epoxy-based BFRP laminates. This trend also follows the differences in tensile modulus of the resin itself, which are 3.2, 1.9 and 4.5 GPa, respectively, for WS, AB and CC.

Considering the rule of mixture and using a Krenchel factor of 0.5 [41] (since half the fibres were aligned with the loading direction for biaxial FRP), the basalt fibre strength can be found within the range of 1330–1575 MPa for the samples tested in this study.

Chen et al. [42] conducted a study on the effect of strain rate on static and dynamic properties of BFRP laminates where unidirectional BFRP of 300 gsm and WEST SYSTEM^®^ 105 (both materials are identical in this study) as epoxy were used to produce 0.7 mm thick laminates using a wet layup process. They reported laminate strength within the range of 268–292 MPa, which is very close to this study (296–347 MPa). This tensile strength of laminate translates to a fibre strength of 1562–1729 MPa, based on the rule of mixture. In another study [41], using biaxial basalt fibre (220 gsm) and polyester as resin, with a volume fraction of 0.3, the tensile strength and modulus were reported as 291 MPa and 14 GPa, respectively. Ranganathan et al. [43] obtained a tensile strength and modulus of 377 MPa and 9.7 GPa, respectively, for six-layer uni-directional BFRP with a volume fraction of 0.216. One study [44] involving carbon fibre and bio resin reported a tensile strength and modulus of 650 MPa and 55 GPa, respectively [31% bio epoxy with a Strength and Young’s modulus of 68.5 MPa and 3000 MPa, respectively]; this higher strength and modulus could be attributed to the higher tensile strength of the epoxy in the study as well as the higher tensile strength that carbon fibre usually possess over basalt fibre [45].

The fibre strength of BFRP varies significantly, depending on its chemical composition and sizing. The basalt fibre tensile strength was reported within the range of 1850–4800 MPa in [46]. Chen et al. [42] conducted a detailed study on the effect of sizing and chemical composition of basalt fibre and reported fibre strength in the range of 1220–2830 depending on the varying chemical composition and sizing.

Most results obtained are comparable to other previous studies involving fibre-reinforced materials. Table 4 Shows the comparison of the tensile strength and modulus results of this study with the values from the previous study.

### 3.2. Open-Hole Tensile Strength

**Figure 7:**
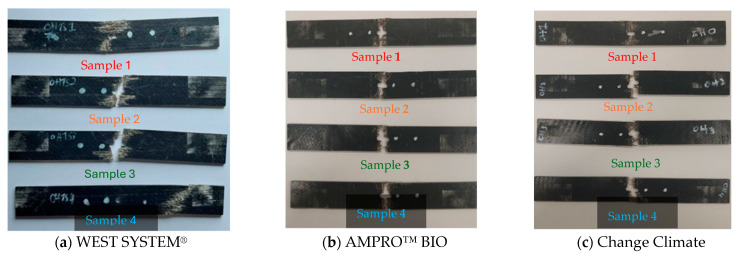
Failure modes of BFRP laminates with various adhesives under open-hole tension test.

The open-hole tensile strength test was conducted to ensure failure within the desired gauge region within the sample. According to ASTM D5766/D5766M–02a, the only acceptable failure mode is the one that occurs near the hole within the specimen. All, except one, specimens failed near the hole, as illustrated in Figure 7.

The only sample that failed around the grip was Sample 4 for WEST SYSTEM^®^, which can be observed in Figure 7a. Due to this reason, this specimen is excluded from the graph and calculations.

**Figure 8:**
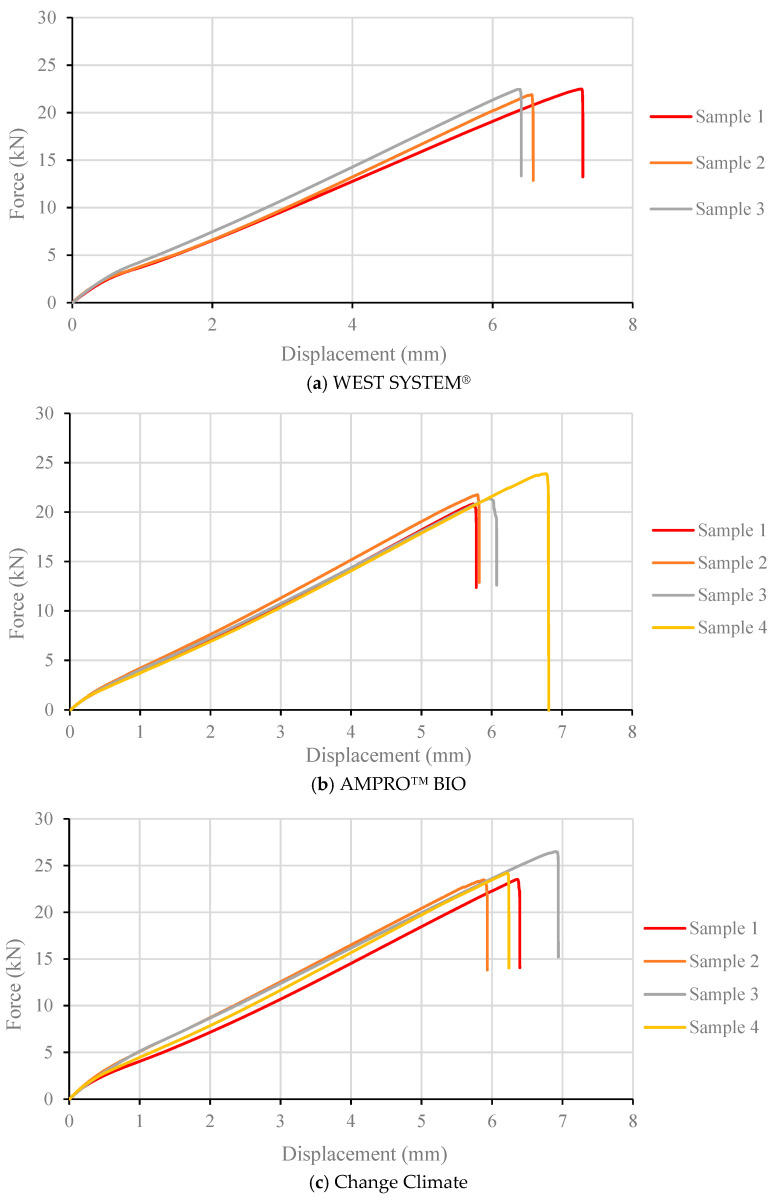
Force vs. displacement curves of BFRP laminates with various adhesives under open-hole tension test.

Figure 8 shows the force vs. displacement curves of all the samples made from the three types of adhesives. The consistencies between samples can clearly be observed. The open-hole tensile strength was determined using Equation (1), and the values are listed in Table 5. CC resin displayed a higher open-hole strength compared to traditional epoxy (by 8%) and AB (by 10%) epoxy. The open-hole strength between traditional and AB resin were similar, with traditional epoxy yielding a slightly higher value. Again, this trend follows the tensile strength of the adhesives’ themselves.

The open-hole-to-pristine-sample-strength ratios are 0.67, 0.62 and 0.62 for WEST SYSTEM^®^, AMPRO™ BIO and Change Climate, respectively. Sun et al. [47] conducted open-hole testing with 6 mm diameter of basalt fibre laminate consisting of one biaxial (0–90) layer using traditional epoxy. They reported a reduction of 44% in the 6 mm-hole-diameter basalt fibre sample compared to the pristine sample. It can be noted that the fibre volume fraction was not mentioned in [47]. Nevertheless, the reduction in peak strength/load in their work and in the present study is comparable. Table 6 shows the open-hole strength of this study compared with the studies conducted previously on the topic. Tuo et al. [48] reported an open-hole strength of 470 MPa for carbon fibre composites made with traditional epoxy [stacking sequence of [45/−45/90/0/−45/0/0/45/−45/0]_s_ was used; these results seem to indicate a much higher open-hole strength compared to the current study, and this is most likely due to the higher tensile strength that carbon fibre possesses when compared with basalt fibre. Depending on the fibre type, carbon fibre could be twice as strong in tensile strength when compared to basalt fibre [45]. Another study [49] which uses glass fibre composites made from vacuum bagging processes reported an open-hole strength of 112.4 MPa [Plain Wolven C glass fibre, 12 layers] which is lower than the results obtained in this study, which correlates with the physical properties of glass fibre, typically placing it below basalt fibres in tensile strength which can change depending on the fibre type used [45]. Some studies involving open-hole tests made with basalt composites can also be found. One such study, Fernandes et al. [50], reports an open-hole strength of 274 MPa for a basalt fibre composite made with the compression moulding method [bidirectional (2-D) basalt with a layup of [0 90]_5_]. This result is within the range of the values obtained during this study, and the slightly high strength of the previous study could be attributed to the extra layer of basalt present in the laminate.

### 3.3. In-Plane Shear

**Figure 9:**
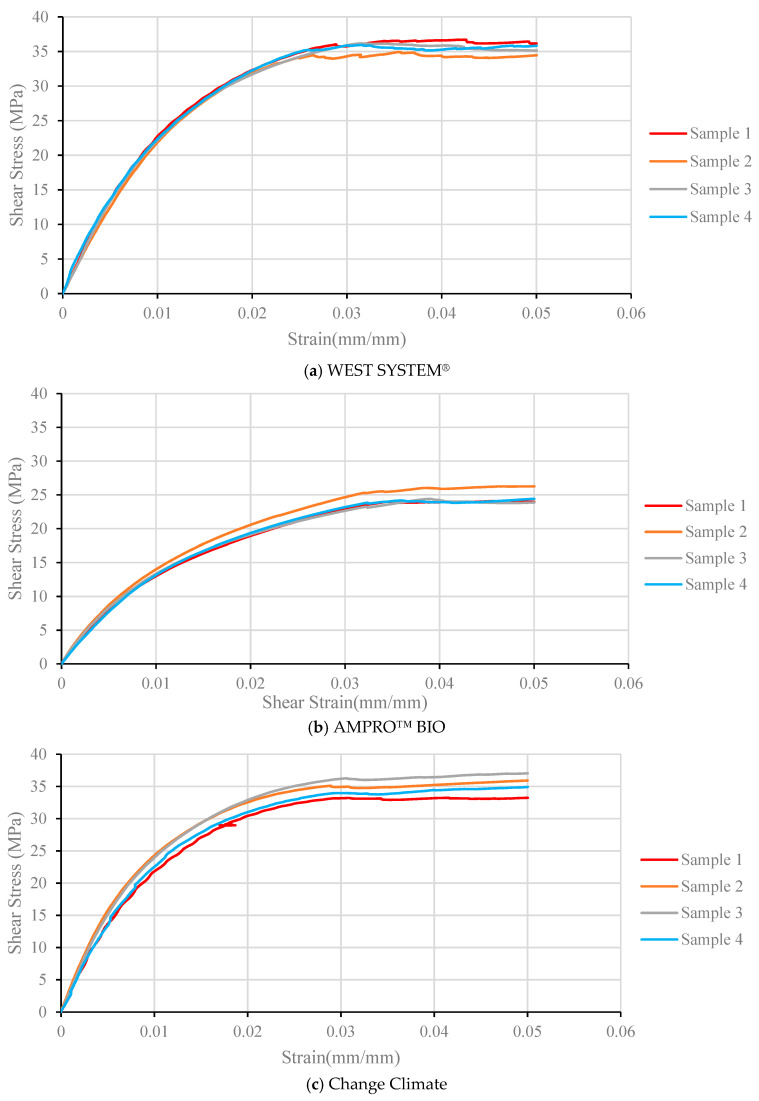
Shear stress vs. strain curves of BFRP laminates with various adhesives.

The in-plane shear properties of the BFRP laminate were determined using tensile tests with the fibres oriented in the ±45 direction. The resin system has significant impact on the shear properties of a laminate, and, hence, this test can directly compare the performance of the traditional vs. bio epoxy on the BFRP laminates. Almost every specimen within the same resin system exhibited similar mechanical properties, as seen in Figure 9.

The Change Climate epoxy was found to have very comparable in-plane properties with traditional epoxy with less than a 5% difference in terms of both shear strength and modulus (Table 7). AMPRO™ BIO, in contrast, attained 69% of the shear strength and 58% of the shear modulus of traditional epoxy. As mentioned earlier, the AMPRO™ BIO epoxy has lower mechanical properties compared to WEST SYSTEM^®^ and Change Climate adhesives.

The in-plane shear strength and modulus of basalt fibre-reinforced epoxies were reported to be 42 MPa and 2.72 GPa, respectively, in [41]. Although these values are comparable to those in Table 7, the fibre volume fraction in [41] was 0.3, and unsaturated polyester was used as the resin. Scalici et al. [27] reported an in-plane shear strength and modulus of 21.7 MPa and 2.08 GPa, respectively, for basalt fibre of 580 gsm (tensile strength and modulus of 2130 MPa and 93 GPa, respectively) and low viscosity resin (tensile strength and modulus of 60 MPa and 2.7 GPa, respectively). The basalt fibre in this study was continuous basalt fibre yarns, plain woven with low tex weft basalt ties. Scalici et al. implemented the vacuum infusion technique and obtained a volume fraction of 0.57. They reported that they were expecting a shear strength of 54 MPa, according to the rule of mixture. Limited studies of fibre reinforced with bio resin were found. Boursier et al. [44] tested carbon fibre composites reinforced with bio resins and reported a shear strength of 61 MPa and a shear modulus of 3 GPa [31% bio epoxy with Strength and Young’s modulus of 68.5 MPa and 3000 MPa, respectively; ASTM D5379 was used]; the higher shear strength compared to this study can be attributed to the superior tensile strength properties of the resin used in the study which is higher than any resin used during this study. Overall, the results obtained in this study agree well with limited studies reported in the literature on the in-plane shear properties of basalt fibre. Table 8 shows the results from other studies with the ones obtained during this study for a better comparison.

## 4. Result and Discussion—Interface Properties

### 4.1. Mode I Fracture Properties

**Figure 10:**
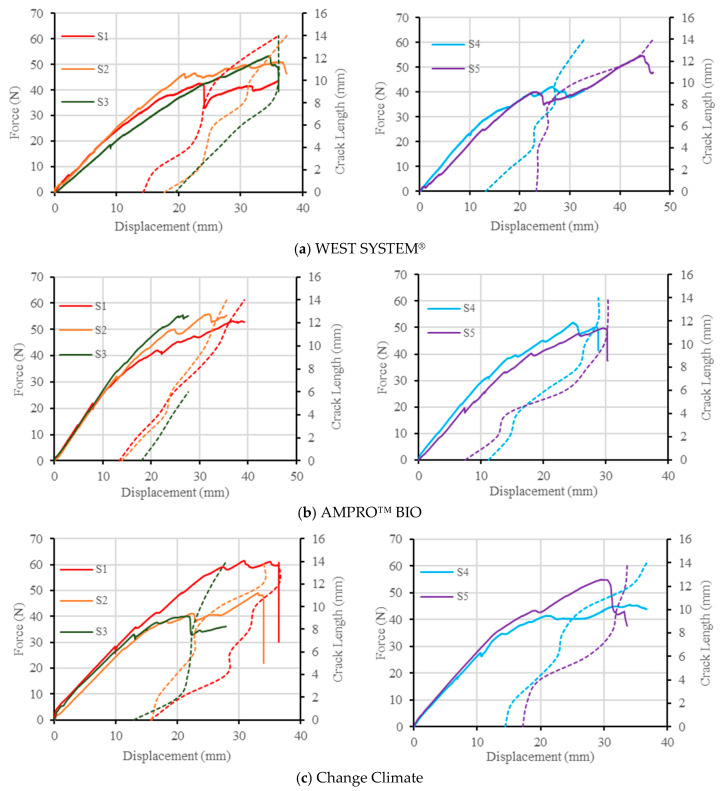
Force vs. displacement vs. crack length graph for all resin systems in mode I testing. The solid lines represent force vs. displacement plots, the dotted lines are the corresponding displacement vs. crack length measurements.

Figure 10 shows the force vs. displacement vs. crack length curves of all the BFRP laminates made from three different types of resins. The solid lines represent force vs. displacement plots, while the dotted lines are related to the corresponding displacement vs. crack length measurements. It is apparent that, irrespective of the adhesive system used in this study, the force vs. displacement behaviour remained similar with WEST SYSTEM^®^, reaching the maximum force within the range of 42–55 N. This range was 50–56 and 40–61 for AMPRO™ BIO and Change Climate, respectively. Non-linearity in the curve or deviation from linearity in the force vs. displacement curve indicate material resistance with respect to crack propagation. Non-linearity started around half the ultimate load which is represented by the change in slope of the force vs. displacement curves. This is a desired behaviour which was noticed in all the resin systems.

**Figure 11:**
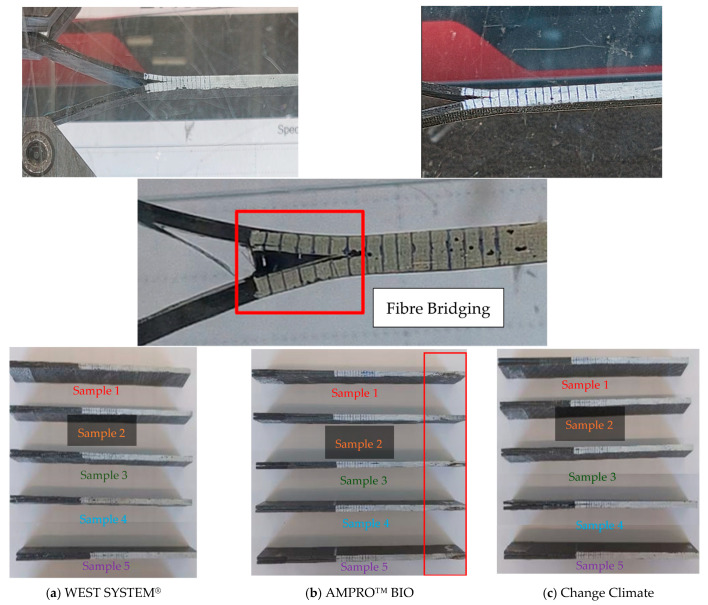
Typical failure mode observed during mode I testing.

All the samples failed under the desired failure mode, i.e., one crack propagating from the initial crack tip and through the midplane of the beam. Occasionally, slight deviation from the midplane was observed as shown in Figure 11. Samples associated with AMPRO™ BIO resin showed some end failure on the right of the specimen, as highlighted in red in Figure 11b. Such delamination migration was significant in Sample 3 (see Figure 10b), where the test stopped at 27 mm displacement abruptly, resulting in a crack propagation of only 6 mm compared to ~15 mm in other samples.

**Figure 12:**
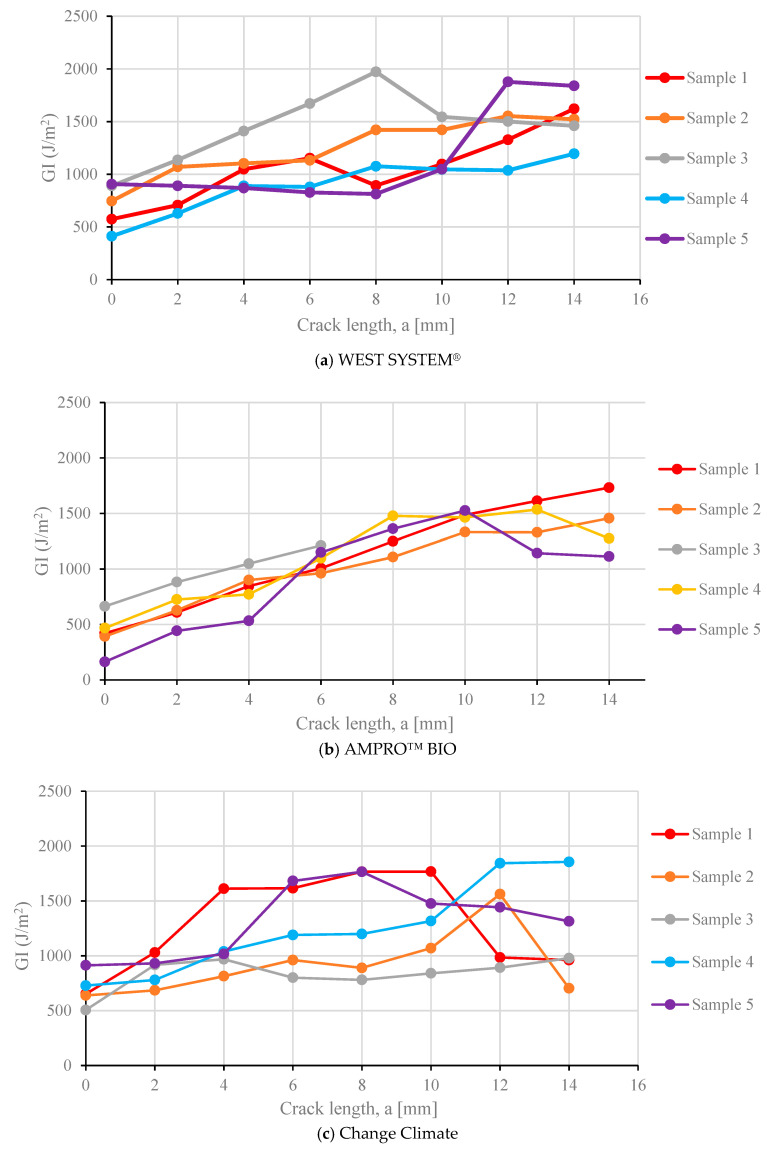
Crack resistance curves (R-Curves) subjected to mode I fracture.

The crack propagation was measured against the 2 mm equidistant vertical lines using a video camera. It was then synchronized with the load vs. displacement plot and then mode I strain energy release rate was calculated using Equation (5). The evaluation of mode I strain energy release rate with respect to crack propagation can be seen in Figure 12 to obtain the resistance curve (R-curve). The WEST SYSTEM^®^ resin covered a broad range of mode I strain energy release rates, starting from 411 J/m^2^ with a max value of 1972 J/m^2^ (Figure 12a). This range was similar for Change Climate, between 506 and 1855 J/m^2^ (Figure 12c). For AMPRO™ BIO, the highest value was 1700 J/m^2^, with a minimum value of 162 J/m^2^ (Figure 12b). Sample 3 only propagated up to 6 mm and the reason behind this was discussed before.

Even though the failure modes observed in this study were satisfactory, unstable crack propagation is apparent in Figure 12. This can be due to various reasons, including fibre bridging shown in Figure 11, intra-laminar delamination, residual stresses or a combination of these [51]. These effects are expected to occur at the interface of the *ϑ*-oriented lamina. For multidirectional laminate, the initiation values are recommended to be reported as G_IC_. Therefore, Table 9 contain both the initiation and average G_IC_.

**Figure 13:**
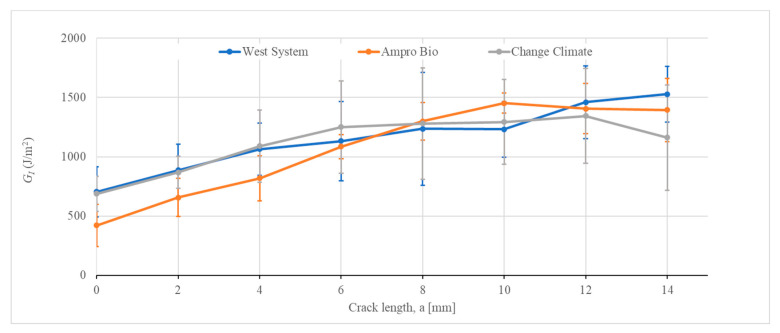
Comparison of mode I R-curve of all three resin systems.

To compare the performance of all the resins, the mean of the strain energy release rate was determined at every 2 mm of crack length and plotted in Figure 13. As the crack propagates, an upward slope in strain energy release rate can be observed. All three resin systems exhibited increased resistance up to 8 mm due to the reasons provided above. At an 8 mm crack length, all resin systems attained similar resistance. This upward slope in the R curve is possibly due to the development of a transverse crack [51].

Beyond an 8 mm crack length, the strain energy release rate remained almost constant up to a crack length of 12 mm, followed by a dropin the release rate for Change Climate resin. For AMPRO™ BIO, the resistance kept increasing until 10 mm, followed by stagnant resistance up to 14 mm. WEST SYSTEM^®^, on the contrary, showed increased resistance till 14 mm crack length. Considering the initiation value as the mode I strain energy release rate, WEST SYSTEM^®^ and Change Climate yielded similar values, with a 2% reduction in the latter compared to the former. However, AMPRO™ BIO showed a 40% reduction in mode I strain energy release rate compared to the traditional epoxy.

In comparison to other fibre-reinforced materials, basalt fibre laminates exhibited comparable properties to other FRPs. Table 9 compares the critical strain energy release rate under mode I fracture in this test with tests conducted previously.

### 4.2. Mode II Fracture Properties

**Figure 14:**
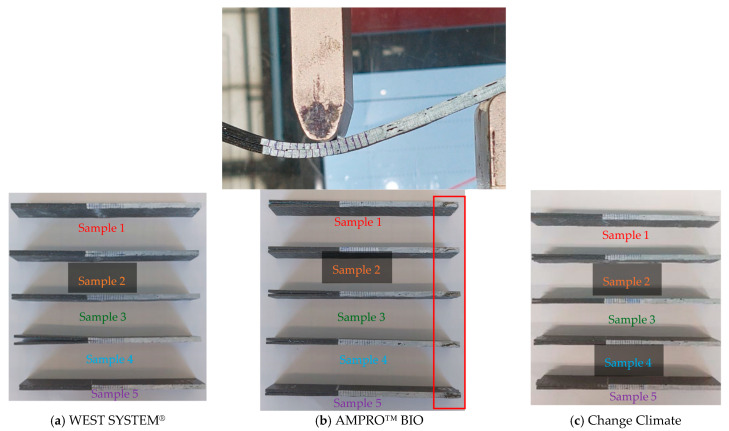
Typical failure mode observed during mode II testing.

Obtaining pure mode II fracture failure mode is often difficult, since cracks may radiate from the supports or from the vicinity of the loading pin due to bearing issues. However, delamination through the midplane along the line of the pre-crack was the mode of failure for all the samples, as shown in Figure 14. Similar to the mode I scenario, local crushing was observed near the support region for the AMPRO™ BIO samples, as displayed in Figure 14b. This could be attributed to lower shear strength and modulus values for AMPRO™ BIO, as reported in Table 7.

**Figure 15:**
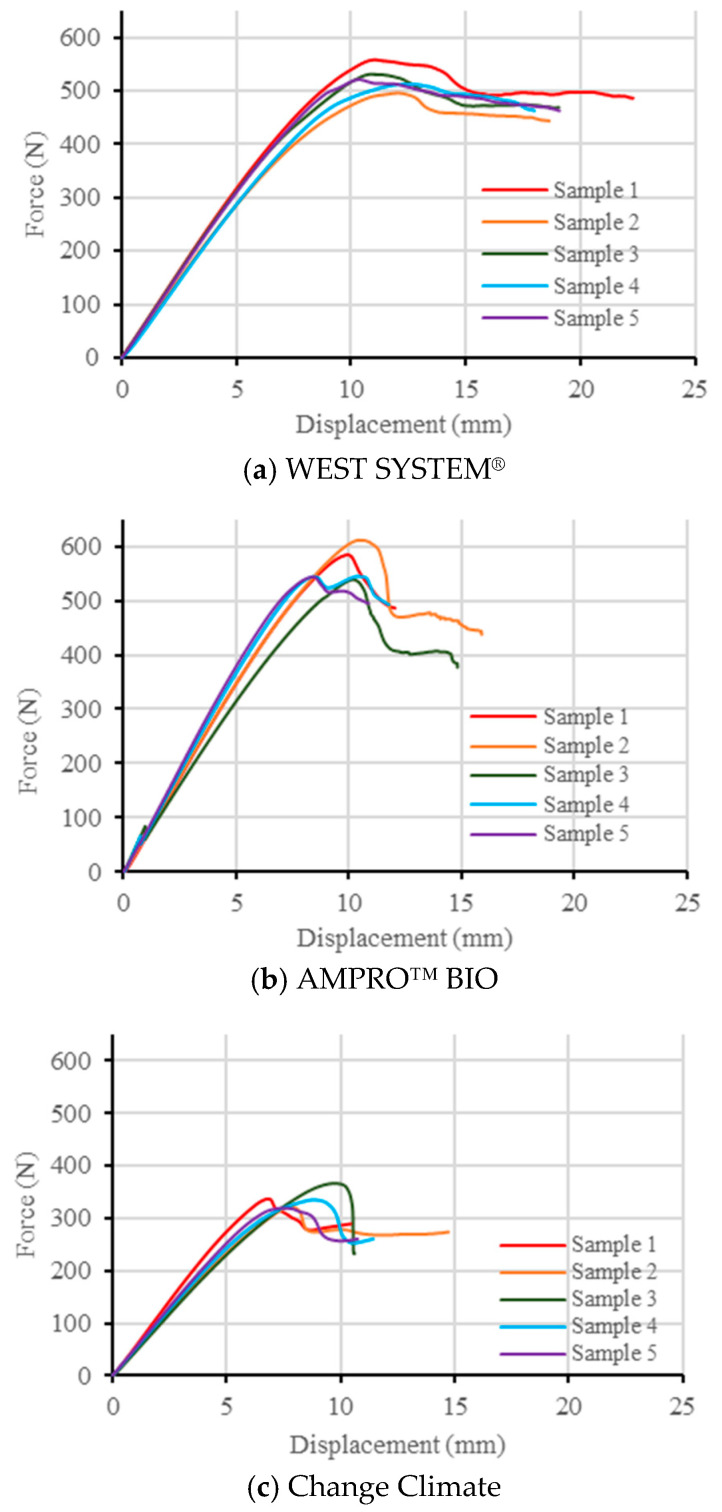
Force vs. displacement vs. crack length graph for all resin systems in mode II testing.

From Figure 15, it can be observed that the load vs. displacement curve remained almost linear until it reached the peak value, followed by a plateau. This plateau was relatively long in WEST SYSTEM^®^, followed by Change Climate. The short plateau in AMPRO™ BIO is possibly due to the local crushing observed near the support region. In terms of ultimate load, both WEST SYSTEM^®^ and AMPRO™ BIO reached similar values (within 500–600 N) compared to Change Climate, which reached an ultimate load less than 350 N. This leads to a lower mode II strain energy release rate for Change Climate, as tabulated in Table 10.

Limited studies were found for G_IIC_ related to basalt fibre. Scalici et al. [27] reported G_IIC.initiation_ 420 N/m for basalt fibre made from vacuum infusion (*v_f_* = 0.57, 580 gsm basalt fabric, [0]_8_ stacking sequence and traditional epoxy). Sriranga et al. [55] reported a GIIC value of 844 N/m for glass/epoxy composites made with S glass fibre 280 gsm and polyester epoxy resin using the vacuum bagging method [8 plies of interwoven glass fibres were used]. In another study involving natural fibres [56], flax composites were made with compression moulding which indicated a GIIC value of 1405 N/m [200 gsm flax fibres, four layers of flax fibres and four layers of unidirectional carbon fibre on the outer surface of flax to avoid mixed crack growth]. The GIIC values found in previous studies, including the one with basalt fibre, all seem to be lower than the results found in this study. The mechanical properties of the resins used in the studies indicate that they exceed the mechanical properties of the resins used in this study [27,57]. This might indicate a stronger bond between basalt fibre and resin systems used in this study. Table 11 shows the results of this study against the ones from other studies.

## 5. Conclusions

The present study examined tensile, shear and fracture properties of basalt fibre laminates made from bio-based resins. Two types of bio-based resins containing 40 and 77% bio-based ingredients were considered, namely AMPRO™ BIO and Change Climate, respectively. Their mechanical and fracture performance were compared against traditional epoxy.

- Both bio resins attained superior tensile strength compared to traditional epoxy by an amount of 6 and 17% for AMPRO™ BIO and Change Climate resins, respectively;
- In terms of tensile modulus, however, the effect of resin was negligible, with Change Climate attaining slightly higher (5%) and AMPRO™ BIO achieving slightly lower (5%) values;
- When it comes to in-plane shear strength and modulus, Change Climate attained similar values compared to traditional epoxy (2% lower strength and 5% higher modulus). AMPRO™ BIO exhibited reduction in shear strength and modulus by 31 and 42%;
- It can be noted that AMPRO™ BIO resin itself had lower tensile properties compared to the traditional epoxy used in this study, whereas Change Climate had similar mechanical properties to its traditional counterpart;
- In Mode I, Change Climate attained a similar value compared to traditional epoxy. Compared to traditional epoxy, there was a 40 and 2% reduction in mode I critical energy release rate for AMPRO™ BIO and Change Climate, respectively;
- In terms of Mode II fracture toughness, however, AMPRO™ BIO was improved by 13% and Change Climate was reduced by 13% compared to the same traditional epoxy. Change climate achieved the lowest in terms of Mode II.

From this study, it can be concluded that the bio-based resin shows promising results in terms of some mechanical and fracture properties. Thus, bio-based resins have the potential to replace traditional epoxies which can make the basalt fibre laminate more sustainable. It is noteworthy to mention that this study did not consider the effect of curing, i.e., curing conditions were kept the same for all three types of adhesives; furthermore, this study only focused on mode I and mode II properties separately. Future studies will investigate cure path dependencies of these epoxies on their mechanical properties as well as the mixed mode failure properties of BFRP.

## Figures and Tables

**Table 1:** Resin-to-hardener ratio by weight and by volume.

Brand Name of Epoxy	Resin Type	Ratio by Weight
WEST SYSTEM^®^ 105/207	Traditional resin	5.36:1
AMPRO™ BIO	40% bio content	3.33:1
Change Climate Bio-Epoxy	77% bio content	3.35:1

**Table 2:** Summary of the tests performed.

Test	ASTM Standard	Mechanical Properties	Sample Tested	References
Standard tension	ASTM D3039/D3039M	Tensile strength and modulus	4	[33]
Open-hole tension	ASTM D5766/D5766M	Open-hole tensile strength	4	[34]
In-plane shear	ASTM D3518/D3156M	In-plane shear strength, modulus	4	[35]
Mode I interlaminar Fracture Toughness	ASTM D5528–13	Mode I interlaminar fracture toughness	5	[36]
Mode II interlaminar Fracture Toughness	ASTM D7905/D7905M–19	Mode II interlaminar fracture toughness	5	[37]

**Table 3:** Tensile strength and modulus of BFRP laminates with various adhesives.

	Tensile Strength (MPa)			Tensile Modulus (GPa)		
	WS	AB	CC	WS	AB	CC
Sample 1	277.5	328.3	385.5	11.15	10.8	13.02
Sample 2	293.1	313.6	352.1	10.28	11.48	11.64
Sample 3	301.8	292.4	360.6	12.93	10.94	11.17
Sample 4	312.5	326.3	291.7	11.79	11.00	12.44
Mean ± SD	296.2 ± 14.8	315.2 ± 16.5	347.5 ± 39.8	11.54 ± 1.12	11.05 ± 0.30	12.07 ± 0.82

WS = WEST SYSTEM^®^, AB = AMPRO™ BIO, CC = Change Climate, SD = Standard deviation.

**Table 4:** Comparison of tensile strength and modulus for various fibre-reinforced polymer.

Article	Fibre	Resin	Tensile Strength (MPa)	Tensile Modulus (GPa)
Soares et al. [41]	Basalt Fibre–220 gsm	Unsaturated Polyester	291.4 ± 6.3%	14.3 ± 7.1%
Chen et al. [42]	Basalt Fibre–300 gsm	WEST SYSTEM^®^	282.4 ± 10.4	N/A
Ranganathan et al. [43]	Basalt Fibre–240 gsm	Vinyl ester resin	377.6 ± 15.3	9.7 ± 0.72
Boursier et al. [44]	Carbon Fibre	31% bio epoxy	650 ± 7.1	54.8 ± 0.14
This study	Basalt	WEST SYSTEM^®^	296.2 ± 14.8	11.54 ± 1.12
This study		AMPRO™ BIO	315.2 ± 16.5	11.05 ± 0.30
This study		Change Climate	347.5 ± 39.8	12.07 ± 0.82

**Table 5:** Open-hole strength of BFRP laminates with various adhesives.

Open-Hole Strength (MPa)			
SP No.	WEST SYSTEM^®^	AMPRO™ BIO	Change Climate
Sample 1	204.3	185.4	210.8
Sample 2	186.0	191.3	208.3
Sample 3	207.2	191.7	232.2
Sample 4	-	214.0	210.1
Mean (±SD)	199.2 ± 11.49	195.6 ± 12.61	215.4 ± 11.3

**Table 6:** Comparison of open-hole strength for various fibre-reinforced polymers.

Article	Fibre	Resin	Open-Hole Strength (MPa)
Tuo et al. [48]	Carbon Fibre	Traditional Epoxy	470.5
Shaari et al. [49]	Plain Wolven C glass fiber	Traditional Epoxy	112.4 ± 7.17
Fernandes et al. [50]	Bidirectional Basalt Fibre	Traditional Epoxy	274.39 ± 16.20
This study	Basalt	WEST SYSTEM^®^	199.2 ± 11.49
This study		AMPRO™ BIO	195.6 ± 12.61
This study		Change Climate	215.4 ± 11.3

**Table 7:** In-plane shear strength and modulus of BFRP laminates with various adhesives.

	Shear Strength (MPa)			Shear Modulus (GPa)		
	WS	AB	CC	WS	AB	CC
Sample 1	36.72	24.08	33.25	2.49	1.35	2.32
Sample 2	34.93	26.26	35.92	2.34	1.45	2.63
Sample 3	36.17	24.40	37.05	2.48	1.39	2.64
Sample 4	35.95	24.41	34.92	2.30	1.39	2.47
Mean ± SD	35.94 ± 0.75	24.79 ± 0.99	35.28 ± 1.61	2.40 ± 0.10	1.39 ± 0.04	2.52 ± 0.15

**Table 8:** Comparison of shear strength and modulus for various fibre-reinforced polymer.

Article	Fibre	Resin	Shear Strength (MPa)	Shear Modulus (GPa)
Boursier et al. [44]	Carbon Fibre	31% Bio epoxy	61.2	3.0 ± 0.1
Soares et al. [41]	Basalt Fibre–220 gsm	Unsaturated polyester	42 ± 12.61%	2.72 ± 9.7%
Scalici et al. [27]	Basalt Fibre–580 gsm	Traditional epoxy	21.7	2.08
This study	Basalt	WEST SYSTEM^®^	35.94 ± 0.75	2.40 ± 0.10
This study		AMPRO™ BIO	24.79 ± 0.99	1.39 ± 0.04
This study		Change Climate	35.28 ± 1.61	2.52 ± 0.15

**Table 9:** Comparison of critical energy release rate for various fibre-reinforced polymers.

Article	Fibre	Resin	Layup	G_IC_ (J/m^2^)
Pereira et al. [52]	Carbon	Traditional epoxy	[0]_24_	369
Zulkifli et al. [53]	Silk	Polyester	N/A	847
Suppakul et al. [54]	E-glass	Vinyl ester	N/A	450
Scalici et al. [27]	Basalt (580 gsm)	Traditional epoxy	N/A	341 (initiation), 596 (propagation)
Zhao et al. [28]	Basalt (350 gsm)	Polycarbonate film	[0 90]_9s_	1224
This study	Basalt	WEST SYSTEM^®^	[0 90]_3s_	705 (initiation) 1155 (average)
This study		AMPRO™ BIO	[0 90]_3s_	421 (initiation) 1066 (average)
This study		Change Climate	[0 90]_3s_	688 (initiation) 1122 (average)

**Table 10:** Critical mode II strain energy release rate G_IIC_ (N/m) for all the resins in this study.

Resin	S1	S2	S3	S4	S5	Mean (±SD)
WEST SYSTEM^®^	2247	1773	2034	1775	1714	1908 (±226)
AMPRO™ BIO	2304	2523	1958	2000	1995	2156 (±248)
Change Climate	1742	1595	1933	1618	1471	1671 (±175)

**Table 11:** Comparison of critical mode II strain energy release rates for various fibre-reinforced polymers.

Article	Fibre	Resin	G_IIC_ (N/m)
Sriranga et al. [55]	S–Glass Fibre	Polyester Epoxy Resin	844
Scalici et al. [27]	Basalt Fibre	Traditional Epoxy Resin	419 (±29)
Prasad et al. [56]	Flax Fibre	Traditional Epoxy Resin	1405 (±31)
This study	Basalt Fibre	WEST SYSTEM^®^	1908 (±226)
This study		AMPRO™ BIO	2156 (±248)
This study		Change Climate	1671 (±175)

## Data Availability

The raw data supporting the conclusions of this article will be made available by the authors on request.
